# The Tiotropium Safety and Performance in Respimat® Trial (TIOSPIR®), a large scale, randomized, controlled, parallel-group trial-design and rationale

**DOI:** 10.1186/1465-9921-14-40

**Published:** 2013-04-02

**Authors:** Robert A Wise, Antonio Anzueto, Peter Calverley, Ronald Dahl, Daniel Dusser, Gordon Pledger, Michael Koenen-Bergmann, Elizabeth Joseph, Daniel Cotton, Bernd Disse

**Affiliations:** 1 Johns Hopkins Asthma & Allergy Center, 5501 Hopkins Bayview Circle, Baltimore, MD, 21224, USA; 2 Pulmonary Critical Care Center, San Antonio, TX, USA; 3 School of Clinical Science, University Hospital Aintree, Liverpool, UK; 4 Department of Respiratory Diseases, Aarhus University Hospital, Aarhus University, Aarhus, Denmark; 5 Service de Pneumologie Hopital Cochin, Paris, France; 6 Hamilton, TX, USA; 7 Boehringer Ingelheim Pharma GmBH & Co, Ingelheim, Germany; 8 Boehringer Ingelheim Pharmaceuticals Inc, Ridgefield, CT, USA

**Keywords:** Tiotropium, COPD, Respimat® Soft Mist™ Inhaler, HandiHaler®

## Abstract

**Background:**

Tiotropium bromide is an effective therapy for COPD patients. Comparing across programs tiotropium Respimat® Soft Mist™ inhaler was at least as efficacious as tiotropium HandiHaler®, however, concerns have been raised about tiotropium’s safety when given via Respimat®.

**Methods:**

The TIOSPIR® trial (NCT01126437) compares the safety and efficacy of tiotropium Respimat® 5 μg once daily (marketed) and 2.5 μg once daily (investigational) with tiotropium HandiHaler® 18 μ once daily (marketed). The hypotheses to be tested are 1). that tiotropium Respimat® 5 μg once daily and Respimat® 2.5 μg once daily are non-inferior to HandiHaler® in terms of all-cause mortality, and 2). that tiotropium Respimat® 5 μg once daily is superior to HandiHaler® in terms of time to first exacerbation. A spirometry substudy evaluates the bronchodilator efficacy. The trial is a randomized, double-blind, double dummy, event-driven, parallel group study. Participants can use any background treatment for COPD except inhaled anticholinergic agents. The study encompasses a wide range of COPD patients, e.g. patients with stable cardiac diseases including arrhythmia can be included. Clinical sites are international and include both primary care as well as specialists.

**Results:**

To date, over 17,000 participants have been randomized from over 1200 sites in 50 countries with an anticipated treatment duration of 2–3 years.

**Conclusion:**

TIOSPIR® will provide precise estimates of the relative safety and efficacy of the Respimat® and HandiHaler® formulations of tiotropium, assess potential dose-dependence of important outcomes and provide information on the clinical epidemiology of COPD in a large international patient cohort.

## Introduction

Tiotropium is a long-acting antimuscarinic bronchodilator indicated for maintenance bronchodilation in patients with chronic obstructive pulmonary disease (COPD) for once-daily inhalation treatment. Two formulations of tiotropium have been developed: SPIRIVA® HandiHaler® 18 μg once daily, a dry powder inhaler (DPI), received initial approval in 2002, and is now approved in more than 100 countries. SPIRIVA® Respimat® 5 μg once daily Soft Mist™ Inhaler (SMI) was developed as a novel multi-dose aqueous aerosol delivery device. It is approved in more than 85 countries, with initial approval received in 2007 [1].

Tiotropium HandiHaler® 18 μg and Respimat® 5 μg have been developed in separate stand-alone programs. Until now, they have only been directly compared in trials up to 4-week duration. Clinical trials with tiotropium HandiHaler® 18 μg and Respimat® 5 μg once daily have demonstrated similar clinically important improvements in lung function, symptoms, and health-related quality of life. In a prespecified pooled analysis of two 4-week, placebo-controlled, crossover trials, the 5 μg dose of tiotropium Respimat® and tiotropium HandiHaler® 18 μg were both significantly superior to placebo on the primary endpoint of trough forced expiratory volume in 1 second (FEV_1_) response. Tiotropium Respimat® 5 μg was non-inferior to tiotropium HandiHaler® in terms of bronchodilator efficacy and both formulations provided a similar systemic availability [2]. In a 4-week crossover study of 134 Japanese patients with COPD [3] tiotropium Respimat® 5 μg and tiotropium HandiHaler® 18 μg had similar lung function efficacy, safety, and pharmacokinetic (PK) profiles [3]. Taken together, these findings indicate that tiotropium Respimat® 5 μg and tiotropium HandiHaler® 18 μg have similar efficacy, systemic exposure, and safety.

Exacerbation benefits have been observed in both clinical trial programs. In the 1-year trial with tiotropium Respimat® 5 μg, [4] the hazard ratio (HR) for time to COPD exacerbation (tiotropium/placebo) was 0.69 (95% confidence interval [CI]: 0.63–0.77). Comparable results using the largest tiotropium HandiHaler® trial, Understanding Potential Long-term Impacts on Function with Tiotropium (UPLIFT®) demonstrated an HR of 0.86 (95% CI, 0.81–0.91) after 4 years [5]. Cross-study comparisons therefore suggest potentially superior effects on reducing the risk of an exacerbation with the Respimat® formulation; however, there have not been direct comparisons in clinical trials of sufficient size and duration to determine whether there are true differences in exacerbation outcomes.

Mortality has been evaluated in HandiHaler® and Respimat® trials [6,7]. Intention-to-treat mortality has been analyzed for the largest study with HandiHaler® formulation (4 years, 5992 patients in UPLIFT®) [5], for the three, 1-year studies with the Respimat® formulation, and in a further 6-month study [4,8,9]. In the UPLIFT® study the risk of a fatal event was lower with tiotropium treatment (in the HandiHaler® formulation) than with placebo. For the 4-year, protocol-defined study period up to day 1440, among patients for whom vital-status information was available, 921 patients died: 14.4% in the tiotropium group and 16.3% in the placebo group (hazard ratio, 0.87; 95% CI, 0.76 - 0.99) [5]. In a retrospective, pooled analysis of the three 1-year and one 6-month placebo-controlled trials with tiotropium Respimat®, including 6096 patients, a numeric increase in all-cause mortality was seen in patients treated with tiotropium Respimat® 5 μg (68; incidence rate [IR]: 2.64 cases per 100 patient-years) compared with placebo (51; IR: 1.98) showing a rate ratio of 1.33 (95% CI: 0.93–1.92) for the planned treatment period; the excess in mortality was observed in patients with known rhythm disorders [1]. The causes of death were diverse and a causal relationship between the use of tiotropium Respimat® and mortality has not been established. In contrast to pooled analyses on trial-level data of three of these studies, including the 10 μg non-marketed dose, [7,10,11] the pooled analyses based on patient-level data have shown a numeric, but non-statistically significant increase in all-cause mortality [6,9]. The Cochrane Review meta-analysis, thus concluded: “Head-to-head trials comparing the dry powder HandiHaler® to the soft mist Respimat® inhaler are required before firm conclusions can be drawn concerning the difference in mortality rates between the inhalers” [11].

Direct comparison studies of tiotropium HandiHaler® 18 μg with Respimat® 5 μg have been limited to 4-week crossover studies on PK and lung function parameters mentioned above. Therefore, prospective data from a trial of adequate size and duration is the appropriate way to establish whether tiotropium Respimat® 5 μg has comparable safety and efficacy with tiotropium HandiHaler® including rare events, i.e. events of death or exacerbations outcomes. A treatment arm with tiotropium Respimat® 2.5 μg is also included because this dose may be preferred in combination with a long-acting beta-agonist (ongoing clinical development program of a fixed-dose combination).

A recent comprehensive review of meta-analyses and pharmaco-epidemiologic studies have also raised the possibility that mortality is increased by tiotropium delivered by Respimat® [12]. Although it has been theorized that there may be greater system absorption, PK and pharmacodynamic studies have shown similar profiles for the two currently marketed formulations of tiotropium [2,3]. A recent high-resolution PK study has shown a slightly lower peak serum concentration with Respimat® [13]. Thus, at present, there is no clear mechanistic explanation for an increased risk profile for tiotropium Respimat® compared with HandiHaler®. Because of this unresolved controversy, there is a compelling need to conduct a mortality-endpoint driven trial comparing the two inhaler formulations.

The purpose of this manuscript is to describe the rationale and methods for TIOtropium Safety and Performance In Respimat® (TIOSPIR®) trial (NCT01126437), a multinational, double-blind, comparative trial evaluating the safety and efficacy of two doses of tiotropium Respimat® (once-daily 2.5 or 5 μg), with tiotropium HandiHaler® (once-daily 18 μg). The trial is a large-scale, event-driven, non-inferiority trial with two primary endpoints: time to all-cause mortality and COPD exacerbation.

## Methods

### Study design

#### Outcome measures

The first primary endpoint for TIOSPIR® is all-cause mortality. Clinical sites follow all randomized patients for vital status until trial conclusion, including patients who prematurely discontinued study medication, and collect medical information regarding the date and cause of fatal events.

The second primary endpoint for TIOSPIR® is time to first COPD exacerbation. COPD exacerbations are defined as a complex of two or more respiratory symptoms (worsening dyspnea, cough, sputum production, chest tightness, or wheezing) related to the underlying COPD, with duration of 3 days or more, that require a change in treatment. Treatment changes include acute treatment with antibiotics and/or steroids, or an addition of new maintenance bronchodilator. Mild exacerbations are those that require new prescription of bronchodilator only, moderate exacerbations are those that require antibiotics and/or systemic corticosteroids without hospitalization, and severe exacerbations are those that lead to hospitalization. The onset date of exacerbations is defined by the onset of the first recorded symptom. Exacerbations that occur within 7 days of onset of a prior exacerbation are counted as a single exacerbation.

Secondary outcome measures will also be analyzed by treatment group to support inferences derived from the primary analysis. These measures include the number of COPD exacerbations, the time to first exacerbation associated with hospitalization, the number of exacerbations associated with hospitalization, and the time to the first major adverse cardiovascular event (e.g. fatal event in system organ classes cardiac and vascular disorders, myocardial infarction [MI], or stroke). Criteria for definition of major adverse cardiovascular events are specified in the protocol and are adjudicated by the site investigator who is masked to the study treatment (see Appendix). In a subset of approximately 1300 patients, spirometry was conducted at baseline and is repeated every 24 weeks. Patients in the substudy perform pulmonary function tests at a single time point in triplicate to record trough FEV_1_ and forced vital capacity (FVC). Tertiary analyses of treatment groups will be performed on subgroups specified by demographic variables, co-morbid conditions, disease severity, and concomitant medications.

#### Treatment arms

**Figure 1:**
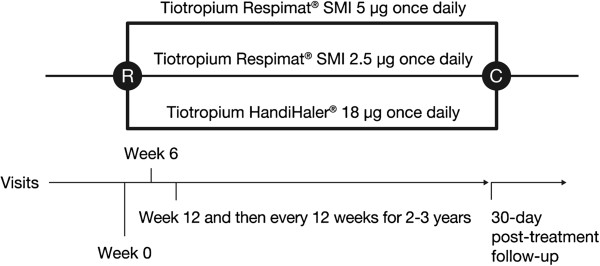
**TIOSPIR® study design.** R, randomization; C, Close of study once the planned 1266 events have been reached; SMI, Soft Mist™ Inhaler. For patients who discontinue trial medication before the planned end of the trial, vital status follow-up continues every 12 weeks.

The study consists of three parallel treatment arms: tiotropium 18 μg once daily via HandiHaler®, tiotropium 5 μg once daily (2.5 μg × two inhalations) via Respimat®, and tiotropium 2.5 μg once daily (1.25 μg × two inhalations) via Respimat® (Figure 1). Each participant was provided with two inhalers, Respimat® and HandiHaler®, one of which contained active medication and the other contained placebo (double dummy). All patients will be followed up for vital status until the end of the study, even if treatment is discontinued prematurely. The planned treatment time for patients will be between 2 and 3 years and is dependent on the number of fatal events observed with study completion expected in 2013. At each 12-week visit, inhalers are collected and new inhalers are distributed. Adherence with drug treatment is ascertained by counting unused HandiHaler® capsules and recording the remaining doses on the Respimat® dose-counter. Proper use of the inhalers is ascertained by direct observation at the first study visit and is maintained by counseling and demonstration, if needed, at subsequent study visits.

The eligibility screening visit may be the same day as the enrollment visit unless it is necessary to acquire off-site source documents. Participants are randomly assigned to one of the three treatment arms with equal probability using an interactive voice or web response system. Each patient is allocated a treatment box containing a unique medication number. The randomization schema is stratified by clinical center using a permuted block design with concealment of subsequent allocation.

### Concomitant medication

All classes of maintenance respiratory medications and medications for other diseases including cardiac diseases and arrhythmia are permitted throughout this trial. Only non-study drug inhaled antimuscarinics (anticholinergics) need to be discontinued.

### Participant selection

#### Inclusion criteria

The eligibility criteria are similar to those in previous late development or post-approval tiotropium HandiHaler® and Respimat® clinical trials [5]. Inclusion criteria are: men or women aged 40 years or older with at least 10 pack-years of smoking, a clinical diagnosis of COPD, post-bronchodilator spirometry showing a FEV_1_/FVC ratio ≤0.70, and a FEV_1_ ≤70% of predicted. Except for those included in the pulmonary function substudy, pre- and post- bronchodilator spirometry performed within 6 months of enrollment could be acquired from patient records. Participants must also demonstrate ability to use both the HandiHaler® and Respimat® devices.

#### Exclusion criteria

Exclusion criteria were rather liberal to reflect a typical COPD population: Participants were excluded if they have significant diseases other than COPD that put the patient at risk for participation in the study or that might interfere with participation in the study. Patients with concomitant cardiac disease could be included unless they had: MI within 6 months, hospitalization for heart failure within 12 months, and unstable or life-threatening arrhythmia requiring intervention or change in drug therapy within 12 months. Patients were also excluded if they had other significant lung diseases, such as active tuberculosis, asthma, cystic fibrosis, clinically evident bronchiectasis, interstitial lung disease, resectional lung surgery, or pulmonary thromboembolism. Patients with cancer (other than skin basal cell) requiring therapy within 5 years, drug or alcohol abuse within 12 months, known hypersensitivity to tiotropium, narrow-angle glaucoma, symptomatic bladder-neck obstruction, pregnancy, lactation, or child-bearing potential not on contraceptives were excluded. Patients were not excluded for radiographic evidence of bronchiectasis if they were not being treated for this condition. Participants with unstable COPD evidenced by an exacerbation within 4 weeks, plans for lung transplantation or lung volume reduction surgery, use of oxygen for more than 12 hours/day, completion of a pulmonary rehabilitation program within 6 weeks of screening, or an unstable dose of systemic corticosteroid medication or chronic use of corticosteroid medication more than the equivalent of 10 mg per day of prednisone were excluded. Participants were also not permitted to participate in other intervention trials or have used an investigational drug within 30 days of enrollment.

#### Study organization and oversight

The scientific oversight of the trial is provided by a Scientific Steering Committee (SSC), which is responsible for making recommendations to the sponsor regarding study design, execution, interpretation, and publication of results. The SSC meets every 6 months to review both the progress and blinded study data. A separate and independent Data Monitoring Committee (DMC) reviews listings and tabulations of adverse events and deaths by treatment group (unblinded if requested) and makes recommendations to the sponsor about continuation of the trial or modifications that are necessary to protect the safety of the participants. The DMC meets every 4 months or more frequently as needed. A Mortality Adjudication Committee (MAC), masked to treatment assignment, is charged with reviewing medical documentation, case report forms, and witness accounts for all deaths in order to establish an attributable cause of death.

The protocol was approved by the respective institutional review boards. The trial is conducted in compliance with the protocol, the principles laid down in the Declaration of Helsinki, in accordance with the ICH Harmonised Tripartite Guideline for Good Clinical Practice (GCP) and in accordance with applicable regulatory requirements. All patients provided written informed consent prior to participation.

### Statistical design

The event-driven trial is designed to end when approximately 1266 deaths are reported. Based on expected mortality, the duration of the trial was estimated to be 3.5 years with 1.5 years enrollment period. The primary mortality analysis is to be conducted using all patients who were assigned treatment and took at least one dose of study drug, regardless of subsequent premature discontinuation of trial. Patients lost to follow-up are censored at the time of last known vital status. For the primary exacerbation analysis, patients are censored at the time of study medication discontinuation.

Mortality is the primary design variable that dictates sample size. The primary analysis includes both mortality and time to first COPD exacerbation, evaluated with the Cox proportional hazard model (with no covariate adjustment) using a hierarchical analysis scheme. This scheme requires that each null hypothesis be tested and rejected before proceeding to the next hypothesis to preserve the trial type I statistical error. The comparisons are to be tested in the following order compared with the HandiHaler® treatment group: 1) non-inferiority for time to death for Respimat® 5 μg; 2) non-inferiority for time to death for Respimat® 2.5 μg; 3) superiority for time to first COPD exacerbation for 5 μg Respimat®. The margin of non-inferiority is set at 1.25 (HR) based on practical considerations, i.e., if the upper bound of the 95% CI for the HR between Respimat® and HandiHaler® lies below 1.25, the null hypothesis is rejected.

### Sample size and power

Assuming a one-sided p-value of 0.025, power of 90% and a non-inferiority margin of 1.25, the total number of events is estimated to be 1266 for the three groups. Assuming a 1.5-year accrual and a maximum follow-up of 3.5 years with 99% ascertainment of vital status, and a 5.6% rate of mortality at 2 years, a total of 16,800 participants (5600 per treatment group) are required.

With this sample size, assuming a two-sided p-value of 5%, and a 35% treatment discontinuation rate with 60% exacerbation rate in the HandiHaler® group, the trial is able to detect an 8% reduction in the HR for first exacerbation with 90% power.

## Results

### Study progress

To train site investigators and staff, a total of 17 training meetings were conducted over a 6-month period at the initiation of the trial.

A total of 17,182 participants were randomized to treatment in more than 1200 investigator sites in 50 countries. Of these, 17,135 participants (99.7%) received at least one dose of medication, constituting the study population.

The trial was initiated in May 2010; enrollment was faster than expected and was completed in April 2011. Because of the quick enrollment, it is likely that the trial duration may be closer to 3 years than the originally expected 3.5 years. Final data collection for the primary outcome measure is anticipated in 2013.

## Discussion

The TIOSPIR® trial has several unique characteristics and presents challenges for the investigators. This is one of the largest COPD trials ever performed, and is being conducted on an international scope. The large size of the trial is dictated by two factors. First is the size of the study population required to assess mortality as an outcome measure, which is an uncommon event in ambulatory patients with COPD who are not oxygen-dependent [14]. Second, the primary outcome analysis is being conducted with a non-inferiority design comparing only active treatments. Thus, this trial is designed to provide a precise estimate of mortality rates as well as exacerbation rates. Another unique feature of this trial is that it compares the same active treatment tiotropium in different marketed delivery systems, formulations and doses to establish the impact of delivery system or dose on clinically rare outcomes, mortality and exacerbations.

This trial has many of the characteristics of a large-scale comparative effectiveness trial: all of the patients are taking active treatments; the inclusion criteria are broad and the study sites are widely representative of both academic and community practices; the outcome measures, mortality and exacerbation rates, are clinically relevant and have important economic implications; and participants are permitted to use their usual background treatments for COPD that do not replicate the class of drug used in the trial. Placebo is not included with the purpose of eliminating a differential drop-out bias, which can be induced by more exacerbations, worse symptoms, and possibly reduced life expectancy. The absence of a placebo group may also facilitate follow-up and drug adherence during a trial of relatively long duration. The duration of the trial (patients are be on treatment for between 2 and 3.5 years) allows for long-term data to be collected on safety and efficacy, including spirometry in the substudy. Thus, TIOSPIR® provides clinically useful information about the optimal use of tiotropium in COPD and guides clinicians, policy makers, and payers. Beyond that, the TIOSPIR® trial can provide information as to the validity of spirometry as a surrogate marker or predictor of efficacy in rare outcomes such as exacerbations.

Despite the organizational and logistic challenges of such a large trial, accrual of participants was faster than expected. This reflected the broad inclusion criteria that were designed to enroll patients who might be encountered in routine clinical practice. Patients with a medical history including MI, cardiac arrhythmia, or cardiac failure were generally included and only excluded if MI occurred within 6 months or less, if cardiac arrhythmia was unstable or life threatening, requiring intervention or a change in drug therapy during the past year, or if cardiac failure of New York Heart Association Class III or IV led to hospitalization during the past year. Moreover, all participants were assigned to an active treatment arm and were permitted to continue other background medications that did not interfere with study participation, and could participate in their usual care routine. One of the challenges in training the large number of investigators, many of whom were community practitioners, was to emphasize the statistical underpinnings of the trial, and the need for high levels of follow up whether or not patients remained on study drug. The study organization and oversight is similar to that used in other large multicenter trials and is designed to provide continuing monitoring and review of the data integrity and patient safety at several levels. This includes an SSC, a DMC, and a MAC that represent the international scope of the trial.

## Conclusions

In summary, the TIOSPIR® trial is designed as a large-scale international trial to ascertain the relative safety and efficacy of different formulations and doses of tiotropium in patients with COPD, based on mortality as the endpoint of highest relevance to clinical outcomes and patient safety, and to concomitantly determine the relative efficacy in preventing COPD exacerbations that are of high relevance to clinical outcomes and cost burden of COPD. This trial requires unique logistic, design, and statistical considerations that differ from smaller trials designed to measure bronchodilator efficacy. Moreover, this large cohort of COPD patients can provide important insights into the clinical epidemiology of COPD around the world.

## Appendix

Major Adverse Cardiovascular Events include the following:

· Stroke

· Transient Ischemic Attack

· Sudden Cardiac Death

· Myocardial Infarction

### Definitions of Major Adverse Cardiovascular Events

#### Stroke

Stroke is defined as an acute onset of focal neurological deficit of presumed vascular origin lasting for 24 hours or more or resulting in death. Additionally, an event lasting >24 hours is considered as a stroke if this is due to 1) therapeutic intervention by pharmacological or non-pharmacological means (i.e., thrombolytics, intracranial angioplasty), or 2) brain imaging available documents a new hemorrhage or infarct. The stroke is categorized as ischemic or hemorrhagic (based on imaging or autopsy) or type unknown. Fatal stroke is defined as death from any cause within 30 days of stroke.

#### Transient Ischemic Attacks (TIA)

A TIA is defined as a rapid onset of a focal neurological deficit that resolves spontaneously without evidence of residual symptoms at 24 hours.

#### Acute Myocardial Infarction

Any one of the following criteria meets the diagnosis for myocardial infarction (MI).

1. Detection of elevated values of cardiac biomarkers (preferably troponin T or I) above the 99th percentile of the upper reference limit (URL) together with evidence of myocardial ischemia with at least one of the following:

· Ischemic symptoms;

· ECG changes indicative of new ischemia (new ST-T changes or new left bundle branch block [LBBB]);

· Development of pathological Q waves in the ECG;

· Imaging evidence of new loss of viable myocardium or new regional wall motion abnormality.

2. Sudden unexpected cardiac death, including cardiac arrest, with symptoms suggestive of myocardial ischemia, or accompanied by new ST elevation, or new LBBB, or definite new thrombus by coronary angiography but dying before blood samples could be obtained, or in the lag phase of cardiac biomarkers in the blood.

3. For percutaneous coronary intervention (PCI) in patients with normal baseline values, elevations of cardiac biomarkers above 99th percentile of the URL are indicative of peri-procedural myocardial necrosis.

4. For coronary artery bypass graft (CABG) in patients with normal baseline values, elevations of cardiac biomarkers above the 99th percentile of the URL are indicative of peri-procedural myocardial necrosis. By convention, increases of biomarkers greater than 5 × 99th percentile of the URL plus either new pathological Q waves or new LBBB, or angiographically documented new graft or native coronary artery occlusion, or imaging evidence of new loss of viable myocardium have been designated as defining CABG-related MI.

5. Pathological findings post-mortem of an acute MI.

## Abbreviations

COPD: Chronic obstructive pulmonary disease; CI: Confidence interval; DMC: Data Monitoring Committee; DPI: Dry powder inhaler; FEV_1_: Forced expiratory volume in 1 second; FVC: Forced vital capacity; HR: Hazard ratio; IR: Incidence rate; MAC: Mortality Adjudication Committee; MI: Myocardial infarction; PK: Pharmacokinetic; SMI: Soft Mist™ Inhaler; SSC: Scientific Steering Committee; TIOSPIR®: TIOtropium Safety and Performance In Respimat®; UPLIFT®: Understanding Potential Long-term Impacts on Function with Tiotropium.

## Competing interests

RW is a paid consultant to Boehringer Ingelheim, Bristol-Myers-Squibb, GlaxoSmithKline, Mylan, Sunovion, Pulmonx, Spiration, and Intermune; the terms of these arrangements are being managed by the Johns Hopkins University in accordance with its conflict of interest policies. AA has received support for research and fees for consultations, travelling and speaking from Boehringer Ingelheim, Forest Labs, GlaxoSmithKline and AstraZeneca. RD has received funding for research and fees for lectures and consultations from Boehringer Ingelheim and Novartis. DD has received fees for consultations, lectures and educational activities from Boehringer Ingelheim, Pfizer, Novartis, Chiesi, Nycomed and Dey Pharma LP. PC has received consultancy, speaking and travelling fees from Novartis, Pfizer and Boehringer Ingelheim. GP provided statistical services under a consulting agreement with Boehringer Ingelheim. MK-B, BD, EJ, and DC are all employees of Boehringer Ingelheim.

## Authors’ contributions

RW, AA, RD, DD, PC, GP, MK-B, BD, EJ and DC all contributed to the study design, data interpretation and development of the manuscript. All of the authors read and approved the final manuscript.
